# Sampling and detection of African swine fever virus within a feed manufacturing and swine production system

**DOI:** 10.1111/tbed.14335

**Published:** 2021-10-06

**Authors:** Jordan T. Gebhardt, Steve S. Dritz, C. Grace Elijah, Cassandra K. Jones, Chad B. Paulk, Jason C. Woodworth

**Affiliations:** ^1^ Department of Diagnostic Medicine/Pathobiology College of Veterinary Medicine Kansas State University Manhattan Kansas USA; ^2^ PIC Hendersonville Tennessee USA; ^3^ Department of Animal Sciences and Industry College of Agriculture Kansas State University Manhattan Kansas USA; ^4^ Department of Grain Science and Industry College of Agriculture Kansas State University Manhattan Kansas USA

**Keywords:** African swine fever virus, epidemiology, swine, viruses

## Abstract

Transmission of biological hazards capable of causing disease in livestock can occur through a wide variety of direct and indirect routes. In swine production, there are a large number of possible routes of exposure of a pathogen into a susceptible population. African swine fever virus (ASFV) has been a significant challenge for Southeast Asia since first detected in China in 2018 and has spread through many countries within the region. In order to understand potential transmission pathways within an ASFV endemic region, a diagnostic investigation was performed to determine the level of contamination on a wide variety of surface types within a live animal production, feed manufacturing, and feed distribution system located in Vietnam. All diagnostic testing was performed locally by the production system's internal diagnostic laboratory using real‐time polymerase chain reaction (rt‐PCR) analysis. Early in the diagnostic investigation, it became clear that feed trucks were a common site of ASFV surface contamination detection. This information resulted in biosecurity‐focused actions for feed trucks arriving back at the feed mill, including decontamination of interior truck cab surfaces and washing of exterior truck surfaces with high‐pressure water prior to application of surface disinfectants. Additionally, a low number of rt‐PCR positive samples were detected within the feed production system, with the greatest number coming from transient surfaces such as high traffic areas and worker clothing. This illustrates the importance of managing employee traffic through procedures such as zoning and separation between clean–dirty areas to reduce the likelihood of pathogen transmission. In conclusion, this report illustrates the importance of routine data capture regarding efficacy of biosecurity procedures which allows for real‐time updates and improvement as biosecurity gaps are identified.

## 1 INTRODUCTION

African swine fever virus (ASFV) is a major challenge for the global swine production industry. The virus is a member of the Asfarviridae family (Galindo & Alonso, 2016), and is relatively large in size compared to many other viruses. It is a double‐stranded DNA virus with a viral envelope consisting of a lipid bilayer membrane (Galindo & Alonso, 2016). To date, there is no efficacious vaccine approved and available for ASFV. Several outbreaks have been reported in recent decades, notably in Georgia in 2007 and China in 2018. Following the introduction into China in 2018, ASFV rapidly spread throughout Southeast Asia. Since ASFV was first introduced into Vietnam in February 2019, it quickly spread to all provinces and urban administrative districts within the country. Information regarding the number of animals culled due to ASFV is limited, but reports in excess of 6 million animals have been provided (FAO, 2021). Pathogens causing swine disease are known to be transmitted via a wide variety of direct and indirect pathways. This is true for ASFV, and multiple transmission pathways have been described including direct pig to pig contact, consumption of contaminated food products commonly known as swill feeding, or other fomites such as vehicles, workers, and other equipment (Bellini et al., 2016; Guinat et al., 2016; Olesen et al., 2020). Once potential pathways of disease transmission have been identified, corrective measures can be put in place to reduce the likelihood of transmission. This is the foundation of biosecurity, and routine monitoring for presence of an infectious agent can help identify potential gaps in biosecurity practices. However, gaps in knowledge currently exist involving the epidemiology of ASFV transmission, particularly through the supply of swine feed and transportation biosecurity. Therefore, the objective of the current investigation was to monitor for the presence of ASFV within a live swine production, feed manufacturing, and distribution system located in an area of ongoing ASFV circulation with the goal of identifying biosecurity gaps so corrective measures can be implemented.

## 2 MATERIALS AND METHODS

### 2.1 Case description

The diagnostic investigation was performed in collaboration with a production company located in Vietnam. This company owns and operates multiple feed manufacturing and live swine production facilities located in some of the most pig‐dense regions of Vietnam. The collaborator is a farrow‐finish producer that incorporates a high level of biosecurity and modern swine production practices including multi‐site production, all‐in/all‐out pig flow, routine decontamination of facilities, and animal transport vehicles between animal groups. As previously indicated, a complex network of transmission pathways exists for ASFV and other swine diseases. Within this investigation, the feed supply chain and live animal marketing station were the primary pathways of interest because the collaborator had the greatest ability to directly incorporate biosecurity improvements to these areas if gaps were identified. The feed supply chain pathway includes a feed mill and associated ingredients, finished feed, surfaces, as well as vehicles for delivery of ingredients and finished feed. Additionally, the production system raises and markets pigs to buyers via a company‐owned market pig transfer station. This facility as well as live market animal transportation vehicles was the second key possible ASFV transmission pathway of interest for this investigation.

Feed for this production system is manufactured by company‐owned feed mills. These feed mills also manufacture and distribute feed to customers outside of their own live animal production. In brief, biosecurity practices at the initiation of sampling employed at the feed mill were external decontamination of truck surfaces (quaternary ammonium, glutaraldehyde, formaldehyde, glyoxal, isopropanol alcohol; CID 20, 1:250 dilution; CID Lines, NV, Leper, Belgium) upon arrival at the facility for all incoming trucks. Trucks were not washed with high‐pressure water, but the disinfectant mist was applied to all incoming vehicles. The disinfectant was allowed to air dry prior to arriving at the finished feed loading area within the feed mill complex. No downtime was required between uses of the trucks and no procedures were in place to disinfect the cabs in the early stages of this investigation. Access to the mill is generally restricted to employees of the company, truck drivers, and management with minimal access permitted to visitors. In the event visitors do enter the facility, they are required to don disposable coveralls, change footwear, and don disposable shoe covers upon arrival at the entry gate to the feed mill. Then, all visitors and employees would walk through a disinfectant mist (CID 20, 1:250 dilution) as they entered the compound. The facility has no formal requirement for downtime away from swine for visitors and personal items such as cellular phones are permitted within the facility with no formal disinfection procedure in place. Facility employees wore company‐provided clothing to and from the facility and there were no clothing change procedures in place upon arrival.

A high percentage of feed manufactured in the facility was pelleted, and a subset of feed manufactured was treated with a commercial formaldehyde‐based feed additive at the labelled dose (SalCURB; Kemin Industries, Des Moines, IA, USA) beginning approximately 1 month prior to initiation of the sampling investigation. Nearly all feed manufactured and distributed from this facility was prepared in bag form (25 kg) and was hand stacked in the back of tarp‐enclosed flatbed trucks for delivery to swine farms. Upon delivery of feed to the swine farms, the exterior surfaces of feed trucks were disinfected at selected sites (sow farms and gilt multiplication growing facilities), but not all sites. Once at the farm, the delivery trucks would park next to feed storage area and the bags were manually unloaded. In the early stages of the sampling investigation, it was determined that feed truck drivers were exiting the trucks at the farm to assist the farm staff unload the bagged feed, although the official policy was for feed truck drivers to remain within the cab of the truck during the unloading process. Feed trucks did deliver feed to known ASFV‐positive sites, and immediately following delivery would be washed and disinfected prior to entering the feed mill complex.

The market animal transfer facility has two separate entrances and there is no cross‐traffic between company‐owned live animal transport vehicles bringing market‐ready pigs to the facility and customer‐owned live animal transport vehicles arriving at the facility to pick up market‐ready animals. The exterior surfaces of all customer‐owned transport vehicles were disinfected when arriving at the market pig transfer station (quaternary ammonium/glutaraldehyde; H.C.G.−150, 1:400 dilution; Mixwell Marketing, Co., Ltd., Bangkok, Thailand). All employees of this transfer station are employees of the herein described production company. Pigs are housed at this facility for a short period of time before being transferred onto customer trucks for delivery to market. The facility is washed and disinfected daily using water followed by application of a commercial disinfectant (H.C.G.−150, 1:400 dilution). No known ASFV‐infected animals, animals from a known ASFV‐infected site, or animals within clinical signs of ASF were transferred to this market animal transfer station.

### 2.2 Sample collection

Throughout the investigation, a variety of samples were collected including (1) feed and feed ingredient samples, (2) surfaces associated with feed manufacture and delivery, (3) surfaces on company‐owned pig transport vehicles, (4) surfaces at a company‐owned transfer station where market pigs are aggregated prior to shipment to customers, and (5) surfaces on customer‐owned pig transport vehicles when arriving at the transfer station to load pigs. Within samples collected from the feed mill environment, feed delivery vehicles, and live animal transport vehicles, a classification scheme was developed to facilitate standardization across sampling locations in terms of risk of surface contamination (Table 1). Zone 1 was defined as surfaces with direct contact with suspected source of contamination (feed, live animal, etc.). Zone 2 was defined as a surface with close proximity (<1 m) or surface with high potential for contamination. Zone 3 was defined as a surface not in close proximity (>1 m) or with potential to become contaminated through fomite transfer. Zone 4 was defined as a fixed location in a non‐production airspace with potential contamination via fomites or non‐fixed surfaces with intermittent contact with possibly contaminated surfaces (brooms, employee footwear, etc.).

**TABLE 1:** Description of sampling zones within location

		Sampling location and respective zoning terminology		
Zone	Definition	Feed mill	Feed trucks	Market animal transfer station
1	Surface with direct/sustained contact with a suspected pathogen transmission entity (feed, animals, faeces, bodily excretion, etc.).	Feed contact surface	Cargo hold	Animal contact surface
2	Surface with a fixed location and close proximity **OR** potential for suspected pathogen transmission entity to be transferred onto surface.	Non‐feed contact <1 m	Exterior	–
3	Surface with a fixed location and moderate proximity within production airspace **OR** potential to contact suspected pathogen transmission entity via fomites within production airspace.	Non‐feed contact >1 m	Interior cab	Interior cab of live animal transport truck, non‐pig contact surface within facility
4	Fixed location surfaces in non‐production airspace with potential of exposure through contact with fomites **OR** non‐fixed location surfaces with intermittent contact with surfaces with potential prior exposure to suspected pathogen transmission entity.	Transient surface	–	Transient surface

Prior to initiation of the sampling investigation, a series of sample size calculations were performed using equations described by Dohoo et al. (2009) using a priori assumptions based on researchers’ previous experience with virus detection surveys in the feed supply chain. Samples types that were prioritized for sample collection based on believed risk of contamination and risk of virus dissemination included areas of high foot traffic, areas where dust can accumulate, and vehicle surfaces given they travel to and from multiple farms. Frequency of sample collection varied throughout the investigation, but the goal at the initiation of the investigation was to conduct weekly sampling to increase understanding of potential gaps in biosecurity. The frequency of sample collection decreased throughout the course of the investigation based on diagnostic laboratory capacity and prioritization considerations and ability for employees to travel to collect samples amid the SARS‐CoV‐2 pandemic. These calculations formed the foundation of the sampling investigation and the sampling strategy was adjusted over the course of the investigation based on ongoing results and as areas of concern were identified and improvements incorporated within the biosecurity program.

Feed and ingredient samples were collected from the feed mill using aseptic technique at multiple sampling locations when logistically feasible using hand‐grab samples of finished feed and ingredients. Finished feed samples were collected directly at the discharge of the finished feed bagging lines, and ingredients from bags or bulk when appropriate. Environmental swabs consisted of 10.2‐cm × 10.2‐cm square gauze sponges that were pre‐moistened with 5 ml phosphate‐buffered saline (PBS) and placed in a clean sealable plastic bag. For each swab sample, the sampling technician used a new sterile glove when handling the swab. At the pre‐determined sampling location, the pre‐moistened swab was used to sample an area approximately 20 × 20 cm^2^ using a crossing pattern where the surface is swabbed vertically to cover area, and then is swabbed horizontally over the same area to ensure appropriate coverage. The swab sample was then placed back in the sealable plastic bag for transportation to the diagnostic laboratory.

### 2.3 Sample analysis

The diagnostic laboratory was owned and operated by the collaborator and was established to perform routine diagnostic testing for the live animal production enterprise. All samples were processed and analyzed locally, and no material whatsoever was transported out of the region. For analysis of feed and ingredient samples, the sampling bag containing the feed or ingredient sample was manually agitated to ensure an even mix, and then the bag was opened aseptically to collect a representative subsample of 5 g of material. The 5‐g subsample was then placed in a sterile container and 25 ml of PBS was added, agitated for 1 h, and the supernatant liquid was collected for real‐time polymerase chain reaction (rt‐PCR) analysis. Environmental swabs were processed by adding 20 ml of PBS to the swab, lightly agitated, and the liquid was collected for rt‐PCR analysis. If samples were not able to be analyzed within approximately 24 h due to high laboratory submission load, samples were placed in −80°C freezer until time of analysis.

All samples were analyzed using a commercially available rt‐PCR assay (VDx ASFV qPCR; Median Diagnostics Inc., Chuncheon, South Korea) designed to detect the p72 viral genome of ASFV following manufacturer recommendations. Amplification was performed using a Stratagene MX3005P qPCR system (Aligent Technologies, Waldbronn, Germany). A total of 40 PCR amplification cycles were used, and if any sample did have detectable ASFV DNA it was considered rt‐PCR positive. If a sample did contain ASFV DNA, values are reported as cycle threshold (Ct).

### 2.4 Statistical analysis

Data were analyzed using linear models using the GLIMMIX procedure in SAS 9.4 (SAS Institute Inc., Cary, NC, USA). Data were categorized in a binary manner based on rt‐PCR result (0 if no detected ASFV DNA, 1 if ASFV DNA detected) and fit using a binary distribution, logit link, Laplace approximation, and ridge‐stabilized Newton–Raphson algorithm as described by Magossi, Bai, et al. (2019) and Magossi, Cernicchiaro, et al. (2019). Fixed effects in the statistical models included sampling zone, sampling month, and the associated interaction. For all models, the interaction term was not significant (*P* > .10) and therefore was excluded from the final model. For each sampling location, the overall percentage of samples that were rt‐PCR positive was calculated using the FREQ procedure. Mean probabilities for rt‐PCR positive status are reported along with 95% confidence limits.

## 3 RESULTS

### 3.1 Feed delivery vehicles

As an initial assessment of the potential detection rate of ASFV DNA, the feed supply chain was selected as the sampling location of importance due to the highly connected structure among farms. For all sampling locations, there was no evidence of sampling zone × month interaction (*P* ≥ .986). During the first set of samples collected (78 samples in August 2019 from feed mill environment, feed ingredients, and feed delivery trucks), there were four samples with detected ASFV contamination (Table 2). All four samples were from the cabs of feed delivery trucks (Table 3), which the mill was not currently decontaminating on a routine basis. Thus, following identification of this biosecurity gap, procedures were immediately changed and improvements were made including incorporating liquid disinfection of truck cabs with a commercially available disinfectant (potassium peroxymonosulfate; Virkon S, 1:100 dilution; Lanxess, Cologne, Germany) using manufacturer‐labelled contact time of 10 min. There were three additional rt‐PCR positive samples in September 2019, with two2 again from the cabs and one from the vehicle wheels. An additional biosecurity procedure was implemented thereafter which used high‐pressure water to remove all organic material from exterior surfaces on all feed trucks returning to the mill, large fans to dry vehicles, and then a final application of exterior disinfectant as had previously been used. This additional step of high‐pressure‐water washing was incorporated in the biosecurity program in order to remove organic material with the goal of increasing efficacy of the disinfectant application. There were no additional rt‐PCR positive samples detected on or within feed delivery trucks in the later stages of this diagnostic investigation. When summarizing by sampling zone, six of the seven rt‐PCR positive samples were from the cab (classified as zone 3) and one was from the wheels of feed delivery trucks (classified as zone 2). Due to the relatively low number of rt‐PCR positive samples (seven out of 1009 total samples, 0.69%), there was no evidence of a difference in percentage positive samples by sampling zone (*P* = .413) or sampling month (*P* = .878).

**TABLE 2:** Real‐time polymerase chain reaction (rt‐PCR) analysis of environmental swabs collected from feed delivery vehicle surfaces collected in a region of active African swine fever virus (ASFV) circulation^a^

Factor	Year	Level	Samples	rt‐PCR positive	Percent positive^b^	95% CI	*P*
Zone^c^	–	1	3	0	0.00	0–100	.413
	–	2	421	1	0.24	0.03–1.67	
	–	3	603	6	1.00	0.45–2.20	
Total	–	–	1027	7	0.68	0.18–1.18	
Month	2019	August	54	4	7.41	2.81–18.15	.878
		September	316	3	0.95	0.31–2.91	
		October	89	0	0.00	0–100	
		November	174	0	0.00	0–100	
		December	–	–	–	–	
	2020	January	–	–	–	–	
		February	20	0	0.00	0–100	
		March	20	0	0.00	0–100	
		April	40	0	0.00	0–100	
		May	44	0	0.00	0–100	
		June	12	0	0.00	0–100	
		July	80	0	0.00	0–100	
		August	40	0	0.00	0–100	
		September	80	0	0.00	0–100	
		October	–	–	–	–	
		November	40	0	0.00	0–100	
		December	–	–	–	–	
	2021	January	18	0	0.00	0–100	
Total	–	–	1027	7	0.68	0.18–1.18	

^a^ Environmental swabs consisted of 10.2‐cm × 10.2‐cm square gauze sponges that were pre‐moistened with 5 ml sterile phosphate‐buffered saline, used to sample an area approximately 20 × 20 cm^2^, then 20 ml of phosphate‐buffered saline was added, lightly agitated, and liquid extracted for ASFV p72 rt‐PCR analysis. Biosecurity practices over the course of the investigation were dynamic, and adjustments were made as gaps in biosecurity were identified. Such examples include additional high‐pressure‐water washing of all external surfaces of feed delivery trucks, liquid disinfection of truck cabs, and re‐evaluating biosecurity practices of feed delivery personnel which were all made in fall 2019.

^b^ Model‐adjusted percentage of samples rt‐PCR positive with 95% confidence limits. Sampling zone × month interaction, *P* = .987.

^c^ Zones defined as: zone 1 = cargo hold; 2 = exterior surface; 3 = interior cab surface.

**TABLE 3:** Detailed description of real‐time polymerase‐chain reaction (rt‐PCR) positive samples collected in a region of active African swine fever virus (ASFV) circulation^a^

Sample month	Description	Zone	Cycle threshold
Feed truck			
August 2019	Cabin – before disinfection	3	35.9
August 2019	Cabin – after disinfection	3	35.0
August 2019	Cabin – before disinfection	3	39.1
August 2019	Cabin – before disinfection	3	34.9
September 2019	Wheels – before disinfection	2	38.8
September 2019	Cabin – before disinfection	3	38.8
September 2019	Cabin – after disinfection	3	38.9
Feed mill			
September 2019	Floor – office	3	38.4
February 2020	Feed line – inside	1	37.3
March 2020	Worker clothing	4	38.2
April 2020	Inside surface of pellet mill	1	39.7
April 2020	Feed line – inside	1	39.0
April 2020	Feed line – outside	2	39.9
April 2020	Feed line – outside	2	38.1
July 2020	Worker clothing	4	38.9
September 2020	Mixer – exterior surface	2	39.5
Feed/ingredients			
May 2020	Finished feed	N/A	36.8
Market pig transfer station			
January 2020	Floor within entrance	3	39.5
January 2020	Table/door handle	3	39.9
January 2020	Floor within high traffic area	3	39.8
December 2020	Live animal holding pen floor	1	39.3
Market pig transfer vehicles			
December 2019	Cabin	3	39.5
December 2019	Cabin	3	39.7
December 2019	Cabin	3	38.1
January 2020	Wheels/cargo hold	1	38.1
January 2020	Cabin	3	38.6
January 2020	Cabin	3	37.8
January 2020	Cabin	3	38.3
September 2020	Cabin	3	36.2
September 2020	Wheels/cargo hold	1	36.4
September 2020	Wheels/cargo hold	1	38.0
September 2020	Wheels/cargo hold	1	35.5
September 2020	Cabin	3	35.9
September 2020	Wheels/cargo hold	1	38.2
September 2020	Cabin	3	38.2
October 2020	Wheels/cargo hold	1	40.0
November 2020	Wheels/cargo hold	1	36.1
November 2020	Wheels/cargo hold	1	39.0
November 2020	Cabin	3	36.5

^a^ Environmental swabs consisted of 10.2‐cm × 10.2‐cm square gauze sponges that were pre‐moistened with 5 ml sterile phosphate‐buffered saline, used to sample an area approximately 20 × 20 cm^2^, then 20 ml of phosphate‐buffered saline was added, lightly agitated, and liquid extracted for ASFV p72 rt‐PCR analysis.

### 3.2 Feed mill environment

There was no evidence of a difference in detection of ASFV contamination between feed contact, non‐feed contact either less than or greater than 1 m from feed contact surfaces, or transient surfaces (*P* = .660). Overall, nine total environmental samples were rt‐PCR positive within the feed mill (total of 1100 total samples; percent positive within sampling zone ranging from 0.40% to 1.22%; Table 4). There also was no evidence that detection of ASFV differed by month (*P* = .983). Within the feed mill, the first rt‐PCR positive sample was found in September 2019 from a high‐traffic surface where truck drivers congregate once returning to the mill. The first feed contact surface with evidence of ASFV contamination was in February 2020, and was followed by two additional rt‐PCR positive samples on feed contact surfaces in April 2020. Worker clothing was identified as a source of contamination in March 2020 and this worker was located in the finished bagged feed handling area and another rt‐PCR positive was detected on worker clothing in July 2020. Due to the concerns identified with possible contamination of clothing and inability to definitively determine the origin of the contamination, a policy was implemented where employees working in the finished feed bag handling area were required to change clothing upon arrival at the mill. The goal of this policy was to help reduce the likelihood of bringing contamination into the facility and in contact with bags of feed which are directly transported to swine farms. The final rt‐PCR positive sample collected from within the feed mill was found on an exterior surface of a mixer in September 2020.

**TABLE 4:** Real‐time polymerase‐chain reaction (rt‐PCR) analysis of environmental swabs collected from feed mill surfaces collected in a region of active African swine fever virus (ASFV) circulation^a^

Factor	Year	Level	Samples	rt‐PCR positive	Percent positive^b^	95% CI	*P *
Zone^c^	–	1	324	3	0.93	0.30–2.83	.660
	–	2	245	3	1.22	0.40–3.73	
	–	3	94	1	1.06	0.15–7.18	
	–	4	496	2	0.40	0.10–1.60	
Total	–	–	1159	9	0.78	0.27–1.28	
Month	2019	August	30	0	0.00	0–100	.983
		September	93	1	1.08	0.15–7.25	
		October	25	0	0.00	0–100	
		November	54	0	0.00	0–100	
		December	–	–	–	–	
	2020	January	–	–	–	–	
		February	58	1	1.72	0.24–11.27	
		March	108	1	0.93	0.13–6.29	
		April	119	4	3.36	1.27–8.62	
		May	62	0	0.00	0–100	
		June	105	0	0.00	0–100	
		July	206	1	0.49	0.07–3.37	
		August	60	0	0.00	0–100	
		September	120	1	0.83	0.12–5.69	
		October	–	–	–	–	
		November	60	0	0.00	0–100	
		December	–	–	–	–	
	2021	January	59	0	0.00	0–100	
Total	–	–	1159	9	0.78	0.27–1.28	

^a^ Environmental swabs consisted of 10.2‐cm × 10.2‐cm square gauze sponges that were pre‐moistened with 5 ml sterile phosphate‐buffered saline, used to sample an area approximately 20 × 20 cm^2^, then 20 ml of phosphate‐buffered saline was added, lightly agitated, and liquid extracted for ASFV p72 rt‐PCR analysis.

^b^ Model‐adjusted percentage of samples rt‐PCR positive with 95% confidence limits. Sampling zone × month interaction, *P* = 1.000.

^c^ Zones defined as: zone 1 = feed contact surface; 2 = non‐feed contact surface <1 m; 3 = non‐feed contact surface >1 m; 4 = transient surface.

### 3.3 Ingredients and finished feed

Overall, only one rt‐PCR positive sample out of 142 samples collected from finished feed, ingredients, or water used in feed processing was found (Table 5). The total number of feed and ingredient samples collected in the current investigation was limited, as it was believed that environmental samples would be more reflective of contamination rate over a longer period of time compared to a point‐in‐time sample of feed or ingredient.

**TABLE 5:** Real‐time polymerase‐chain reaction (rt‐PCR) analysis of finished feed, ingredient, and water samples within a feed mill in a region of active African swine fever virus (ASFV) circulation^a^

Item	Samples	rt‐PCR positive	Percent positive^b^
Finished feed	102	1	0.98
Ingredient	34	0	0.00
Water	6	0	0.00
Total	142	1	0.70 (0.00–2.08)

^a^ Samples of finished feed, feed ingredients, and water used in feed processing were collected, and 5 g of feed or ingredient was diluted with 25 ml of phosphate‐buffered saline, lightly agitated, and the resulting liquid was extracted for ASFV p72 rt‐PCR analysis.

^b^ Statistical model not fit due to low rate of rt‐PCR positives. Total percentage rt‐PCR positive reported with 95% confidence interval.

### 3.4 Market pig transfer station

In addition to the feed manufacture and distribution system, the collaborating production company was interested in evaluating the potential for distribution of ASFV contamination via a company‐owned market pig transfer station. It was believed this location posed a risk for ASFV transmission based on the high degree of centralization with frequent arrival of customer trucks where biosecurity practices could not be enforced to the same extent as company‐owned vehicles. Of all areas sampled during this investigation, the market pig transfer station had the greatest rate of rt‐PCR positive environmental swabs (22 out of 533, 4.13%). There was no evidence of a difference in the percent of samples positive for ASFV DNA based on sampling location (*P* = .438). Of the 22 rt‐PCR positive samples, 18 were from live animal transport vehicles (Table 6). Eight of the rt‐PCR positive samples were from animal contact surfaces within the cargo hold or from wheels, whereas 10 rt‐PCR positive samples were collected from within the truck cabs. Only one rt‐PCR positive sample was collected from an animal contact surface within the transfer facility and three rt‐PCR positive samples were collected from high‐human‐traffic areas including flooring and a table/door handle (Table 7). Detection of ASFV surface contamination over time was relatively consistent, with rt‐PCR positive samples collected in every month of sampling. Together, the results from the market pig transfer station indicate that vehicles are an important potential source of contamination as would be expected given they serve to transport livestock within a region of active ASFV circulation. Furthermore, the cabs of vehicles were commonly contaminated with frequent movement of personnel and are challenging surfaces to decontaminate.

**TABLE 6:** Real‐time polymerase‐chain reaction (rt‐PCR) analysis of environmental swabs collected from live animal transport vehicles at a market pig transfer facility in a region of active African swine fever virus (ASFV) circulation^a^

Factor	Year	Level	Samples	rt‐PCR positive	Percent positive^b^	95% CI	*P *
Zone^c^	–	1	167	8	4.79	2.41–9.31	.629
	–	3	167	10	5.99	3.24–10.79	
Total	–	–	334	18	5.39	2.97–7.81	
Month	2019	August	–	–	–	–	.686
		September	–	–	–	–	
		October	–	–	–	–	
		November	–	–	–	–	
		December	22	3	13.62	4.45–34.89	
	2020	January	54	4	7.41	2.80–18.19	
		February	–	–	–	–	
		March	–	–	–	–	
		April	–	–	–	–	
		May	–	–	–	–	
		June	–	–	–	–	
		July	–	–	–	–	
		August	–	–	–	–	
		September	120	7	5.83	2.80–11.76	
		October	42	1	2.38	0.33–15.15	
		November	56	3	5.36	1.73–15.39	
		December	40	0	0.00	0–100	
	2021	January	–	–	–	–	
Total	–	–	334	18	5.39	2.97–7.81	

^a^ Environmental swabs consisted of 10.2‐cm × 10.2‐cm square gauze sponges that were pre‐moistened with 5 ml sterile phosphate‐buffered saline, used to sample an area approximately 20 × 20 cm^2^, then 20 ml of phosphate‐buffered saline was added, lightly agitated, and liquid extracted for ASFV p72 rt‐PCR analysis.

^b^ Model‐adjusted percentage of samples rt‐PCR positive with 95% confidence limits. Sampling zone × month interaction, *P* = .753.

^c^ Zones defined as: zone 1 = wheels/cargo hold; 3 = interior cab surface.

**TABLE 7:** Real‐time polymerase‐chain reaction (rt‐PCR) analysis of environmental swabs collected from surfaces within market pig transfer facility in a region of active African swine fever virus (ASFV) circulation^a^

Factor	Year	Level	Samples	rt‐PCR positive	Percent positive^b^	95% CI	*P *
Zone^c^	–	1	116	1	0.86	0.12–5.93	.373
	–	3	70	3	4.29	1.38–12.54	
	–	4	13	0	0.00	0–100	
Total	–	–	199	4	2.01	0.06–3.96	
Month	2019	August	–	–	–	–	.995
		September	–	–	–	–	
		October	–	–	–	–	
		November	–	–	–	–	
		December	51	0	0.00	0–100	
	2020	January	62	3	4.84	1.56–14.04	
		February	–	–	–	–	
		March	–	–	–	–	
		April	–	–	–	–	
		May	–	–	–	–	
		June	–	–	–	–	
		July	–	–	–	–	
		August	–	–	–	–	
		September	36	0	0.00	0–100	
		October	20	0	0.00	0–100	
		November	20	0	0.00	0–100	
		December	10	1	10.00	1.37–47.05	
	2021	January	–	–	–	–	
Total	–	–	199	4	2.01	0.06–3.96	

^a^ Environmental swabs consisted of 10.2‐cm × 10.2‐cm square gauze sponges that were pre‐moistened with 5 ml sterile phosphate‐buffered saline, used to sample an area approximately 20 × 20 cm^2^, then 20 ml of phosphate‐buffered saline was added, lightly agitated, and liquid extracted for ASFV p72 rt‐PCR analysis.

^b^ Model‐adjusted percentage of samples rt‐PCR positive with 95% confidence limits. Sampling zone × month interaction, *P* = 1.000.

^c^ Zones defined as: zone 1 = animal contact surface; 3 = non‐pig contact surface; 4 = transient surface.

## 4 DISCUSSION

African swine fever is a major challenge for the global swine industry with serious economic implications not only due to the impact on animal morbidity and mortality, but also the wide‐ranging implications on global trade. ASFV is the sole member of the family Asfarviridae, and is a DNA virus very stable in certain environments such as meat products (Galindo & Alonso, 2016; Gaudreault et al., 2020). Transmission of ASFV has been documented to occur through a wide variety of pathways including direct pig to pig contact, consumption of contaminated food products commonly known as swill feeding, or other indirect forms of transmission such as via vehicles, workers, and other equipment (Bellini et al., 2016; Guinat et al., 2016; Olesen et al., 2020). Additionally, ASFV is unique due to the presence of a sylvatic transmission cycle via the soft tick genus *Ornithodoros* (Galindo & Alonso, 2016). Because of the high degree of viral stability in a number of conditions and wide variety of transmission pathways, control of ASFV is very challenging with historically very limited eradication success.

In the current investigation, our goal was to understand the potential areas within a swine live production, feed manufacturing, and distribution system where detection of ASFV DNA could be found. This sampling approach allows for diagnostic information to be gathered and used to make biosecurity improvements. The swine feed supply is a highly linked system, with one feed mill often serving a large number of farms oftentimes over great geographic distances. Consumption of contaminated swine feed has been documented to be a potential route of transmission for a number of diseases including porcine epidemic diarrhoea virus (PEDV; Aubry et al., 2017; Bowman et al., 2015) and ASFV (Niederwerder et al., 2019). In addition to consumption of contaminated feed, the potential for indirect transmission via fomites associated with feed manufacture and delivery (personnel, vehicles, equipment) serves as an additional source of potential pathogen transmission. Because of the high degree of interconnectedness of the feed supply, the current investigation began by evaluating surfaces associated with the manufacture and delivery of swine feed. Additional information reviewing the principles underlying biosecurity plans for feed mills and review of strategies to reduce pathogen transmission through swine feed can be found elsewhere (Cochrane et al., 2016; Stewart et al., 2020).

Early in the investigation, it was identified that feed delivery truck cabs were a common source of ASFV DNA detection. Although this finding was eye opening to the researchers and production system, it is quite logical given the high degree of personnel movement into and out of these areas and relative difficulty to clean and decontaminate compared to exterior vehicle surfaces. Similar results were observed by Greiner (2016) who found that truck foot pedals were the most common areas within a feed mill where contamination of PEDV or porcine deltacoronavirus could be detected. Furthermore, with ASFV specifically, risk‐based modelling has demonstrated that trucks traveling from regions with ASFV circulation are a potential route of disease introduction into a previously naïve region (Mur et al., 2012). As a result of the detection of ASFV DNA within truck cabs, the production system changed their biosecurity practices and incorporated a liquid disinfection procedure where disinfectant was aerosolized using a portable blower with a mounted tank and application nozzle and directed into the truck cabs upon returning to the feed mill prior to entering the perimeter boundary. The system reviewed procedures for employee traffic associated with the delivery of feed and modified procedures to reduce risk of surface contamination if the farm was in a pre‐clinical stage of disease by instituting a policy where delivery truck drivers do not exit the vehicle. Additionally, the production system incorporated a washing system with high‐pressure water for the exterior surfaces of all feed delivery trucks returning to the feed mill prior to application of the disinfectant mist. These changes in biosecurity procedures illustrate that when diagnostic sampling is planned and executed in a strategic manner, the information generated can be extremely valuable and can help generate improvements in biosecurity.

In addition to the information widely available regarding ASFV stability in meat products over extended periods of time, ASFV DNA has been detected in feed ingredients including dried porcine blood (Wen et al., 2019). Additional research has indicated many of these pathogens can extend for significant periods of time and even remain viable after transoceanic shipment (Dee et al., 2018). Furthermore, it has been documented that pigs can be experimentally infected with ASFV when delivered either via drinking water or via swine feed (Niederwerder et al., 2019). In the current investigation, we focused our sampling efforts on environmental samples from within and immediately surrounding the feed mill. Based on previous research with PEDV, once introduced into a feed manufacturing facility, PEDV becomes widely distributed throughout the facility and can be readily detected through environmental sampling (Schumacher et al., 2017). Additionally, the ability to detect viral RNA within the environment can persist for extended periods of time, and can only be removed through intensive cleaning, disinfection, and thermal processing measures (Huss et al., 2017). Recently, evidence has been generated that indicates ASFV can become widely distributed within a feed mill when manufacturing inoculated feed in a controlled setting (Elijah et al., 2021). Additional research is necessary to further understand these environmental distribution characteristics in a real‐world setting, but initial evidence indicates that environmental sampling for ASFV within a feed manufacturing facility may be a useful diagnostic tool similar to sampling for PEDV.

Of the 142 finished feed, ingredient, and water samples collected from the feed mill, only one contained detectable ASFV DNA. This is consistent with recent results reported by Yan et al. (2020), which indicated ASFV PCR‐positive rates ranging from 0.2% to 1.8% in pooled complete feed and ingredient samples across three feed mills in China. Additionally, these data are in agreement with risk assessments put forth by the United States (USDA, 2019) and European Union (EFSA, 2020), which largely describe a low risk of ASFV entry through plant‐based feeds and ingredients. This experiment reveals a directionality of contamination most likely originated from infected farms and transferred via personnel and vehicles. ASFV is considered endemic in Vietnam, which has a variety of ingredient growing and processing conditions, including roadway drying of grains. Under the conditions of the current investigation with a limited number of ingredient samples, no ingredients were identified as being contaminated. This experiment illustrates that the feed supply chain can play an important role in the transmission of pathogens. Furthermore, these data illustrate that both forward contamination (contaminated ingredients to mill to farm) and backward contamination (contaminated farm to mill) must be considered when developing and implementing biosecurity programs. Additional research is needed to have a greater understanding of the risk and role of forward and backward contamination within the feed supply chain.

When focusing on environmental samples collected at the feed mill in the current investigation, there were only nine of 1159 samples which contained detectable ASFV DNA (0.78%). Data recently published indicate that when using feed manufacturing equipment in a controlled research setting to mix feed inoculated with ASFV, the virus can be found on a number of different surface types even after multiple subsequent batches of feed are mixed (Elijah et al., 2021). Within this recent controlled research, the level of detection of ASFV DNA on feed contact, non‐feed contact, and transient surfaces which were researcher boots was greater than what was observed in the current investigation (10%–100% of PCR reactions having detectable ASFV DNA depending on sampling location and batch compared to 0.78% in current investigation). A wide variety of factors could potentially explain the differences observed including sample collection and analytical technique, but also the underlying quantity of contamination is likely much different which could impact the detection rate. Nonetheless, the data reported by Elijah et al. indicate that environmental sampling is a very practical sample type which can be used to detect environmental contamination after mixing ASFV contaminated feed.

In the current investigation, several key areas of environmental contamination were identified including high‐traffic areas and worker clothing. Likewise, the greatest area for contamination within the live animal transfer infrastructure sampled was live animal transport vehicles, specifically the cabs of these vehicles. When evaluating the semi‐quantitative Ct values, there are very high Ct values near the limit of detection for rt‐PCR assays therefore repeatability is a concern. We acknowledge these assays were developed to detect ASFV DNA in biological tissues of infected animals. However, the detection noted in this study indicates the need for development of assay methods that have lower and more consistent detection limits. In general, these data illustrate that surface contamination with ASFV is possible within a region of ongoing transmission.

The stability of ASFV in the environment is somewhat unknown. There have been reports that environmental survival is relatively short (Olesen et al., 2018), although other field reports indicate a much longer duration of potential infectivity especially in the presence of organic material (Davies et al., 2017; Fischer et al., 2020; Mazur‐Panasiuk & Wozniakowski, 2020). With the limitations of the PCR assay, it is not known whether the detected ASFV DNA would be able to cause infection if introduced to naïve pigs. Regardless of the true duration of viral survival on environmental surfaces, detection of viral DNA on surfaces can indicate that improvements in biosecurity should be made to further reduce the risk of disease transmission. Within this production system, a key component of their biosecurity program was cleaning and disinfection of various surfaces at risk for contamination including vehicles. Information can be found elsewhere which reviews cleaning and disinfection specifically for ASFV (De Lorenzi et al., 2020) as well as disinfectant options registered by the United States Environmental Protection Agency to be used to control ASFV in farm settings (US EPA, 2020).

It is important to consider limitations of the current investigation. One limitation is that there was no measure of sample infectivity. Detection of ASFV DNA via rt‐PCR gives no indication that a sample is infectious. Therefore, it is not possible to infer the likelihood of infection in a naïve population of pigs if either direct or indirect contact is made. There are several advantages of using rt‐PCR compared to other diagnostic assays including the flexible sample types which can be analyzed and rapid turnaround time (Rodriguez et al., 2009). It is possible, however, and we believe extremely useful to use this information to identify potential gaps in biosecurity and implement corrective measures to reduce detection of ASFV DNA. Additionally, it is difficult to ascertain whether the low number of PCR positive samples is due to a true low prevalence of contamination, or issues with sample collection, processing, and molecular diagnostic techniques for these challenging sample types. There is additional information to be learned regarding the challenges associated with extraction of viral genetic material from challenging sample types such as feed and environmental samples, but we believe the current investigation highlights how these methods can be incorporated in the field to aid with biosecurity practice assessment. The use of rt‐PCR tests can serve as a rapid, cost‐effective method to evaluate the efficacy of biosecurity practices and identify potential areas for improvement.

Another limitation of the current study is the nature of the investigation was to identify biosecurity gaps with the goal of implementing corrective improvements in biosecurity. Therefore, the biosecurity practices of the system were adjusted as more data were generated and these data are not true estimates of prevalence of ASFV DNA detection within this system over time. It is also important to consider that changes in ASFV DNA detection over time could be attributed to biosecurity improvements, but also could be due to changes in disease prevalence within the region. This system also incorporated a high level of biosecurity within many areas of live animal production as well as feed manufacture and delivery, which means extrapolation to other situations may not be appropriate or provide an accurate assessment of risk. The feed mill in the current investigation also pellets all diets and uses a commercially available formaldehyde‐based feed product in a subset of manufactured diets. Therefore, it is not possible to extrapolate these results beyond these particular conditions, and the rate of ASFV DNA detection may differ under different conditions.

This investigation has illustrated the value that environmental sampling can bring to a system's biosecurity assessment and protocol development. Environmental sampling can also serve as a method of indicating the source of a disease outbreak and its directionality through fomite transmission. Most feed safety research is based on the theory of forward transmission, with a contaminated ingredient being mixed into feed and causing an initial outbreak in animals. However, the evidence in this case instead suggests the possibility of backward contamination, where an outbreak on the farm led to contamination of feed trucks which transferred the contamination back to the feed mill. In summary, a wide variety of factors must be considered to reduce the likelihood of disease transmission and this information provides baseline understanding in a real‐world setting which can be used as a foundation for future investigations to build upon.

## CONFLICT OF INTEREST

The authors declare no conflict of interest.

## ETHICAL APPROVAL

The authors confirm the ethical policies of the journal have been followed. All samples collected and analyzed in the current report were not derived from live animals and were diagnostic in nature.

## Data Availability

The data that support the findings of this study are available from the corresponding author upon reasonable request.
